# Identification of an Inhibitory Fraction from *Cinnamomum cassia* Against Digestive Lipases

**DOI:** 10.3390/molecules31203737

**Published:** 2026-10-18

**Authors:** Sihem Snini, Jannet Kamoun, Peter Balogun, Michele Fiore, Sébastien Violot, Mohamed Sellami, Sophie Ayciriex, Alexandre Noiriel, Ahmed Aloulou, Abdelkarim Abousalham

**Affiliations:** 1 Université Lyon 1, CNRS, ICBMS, UMR 5246, Villeurbanne, France; sihem.snini@etu.univ-lyon1.fr (S.S.); peter.balogun@etu.univ-lyon1.fr (P.B.); michele.fiore@univ-lyon1.fr (M.F.); sophie.ayciriex@univ-lyon1.fr (S.A.); alexandre.noiriel@univ-lyon1.fr (A.N.); 2 Laboratory of Biochemistry and Enzymatic Engineering of Lipases, National Engineering School of Sfax, University of Sfax, Sfax 3038, Tunisia; jannet.kamoun@enis.tn (J.K.); mohamed.sellami@enis.tn (M.S.); ahmed.aloulou@enis.tn (A.A.); 3 Molecular Microbiology and Structural Biochemistry (MMSB), UMR 5086, CNRS, Université Lyon 1, 7 Passage du Vercors, Cedex 07, F-69367 Lyon, France; sebastien.violot@univ-lyon1.fr; 4 The Lyon Hepatology Institute, IHU EVEREST, F-69424 Lyon, France

**Keywords:** *Cinnamomum cassia*, cinncassiol A 19 glucoside, digestive lipases, inhibition, obesity

## Abstract

Natural inhibitors of digestive lipases offer promising alternatives to conventional obesity medications. This study screened nine medicinal plant extracts, identifying the ethanolic bark extract of *Cinnamomum cassia* as the most potent inhibitor for gastric and pancreatic lipases. Using an integrated approach combining bioactivity-guided fractionation and mass spectrometry, we isolated the highly active fraction, F3-1-1, which exhibited dose- and time-dependent inhibitory effects against human and porcine pancreatic lipases, as well as dog gastric lipase (DGL). Further mass spectrometry and peptide mass fingerprint analysis putatively annotated cinncassiol A-19 glucoside as a bioactive constituent. Notably, this compound was likely bound covalently to the catalytic peptide containing the Ser153 residue in DGL, characterized by a mass shift of +553.10 Da. Molecular docking simulations supported these suggestions, demonstrating stable interactions within the catalytic pocket of DGL through hydrogen and hydrophobic bond interactions. These findings highlight *C. cassia* as a promising source of natural anti-obesity agents.

## 1. Introduction

Obesity is a chronic, multifactorial, and relapsing disease that has emerged as one of the most significant global public health challenges [1]. Characterized by an excess and abnormal accumulation of body fat, obesity is strongly associated with an increased risk of various comorbidities, with a cumulative effect from a young age, correlated with several diseases, including cardiovascular diseases and type 2 diabetes, dyslipidemia, metabolic dysfunction-associated liver disease, hypertension, and several cancers [2]. The prevalence of obesity has increased alarmingly in the past decades across all age groups and has become a global epidemic [3]. In 2022, 43% of adults aged 18 years and over were overweight, of whom 16% were obese [4]. A recent epidemiological study showed that the overall prevalence of obesity among children and adolescents aged 5 to 19 years was 8.5% in 2022, compared to 5% in 2015 [5,6]. Among the geriatric population, the prevalence has also increased considerably, reaching 25.3% globally [7]. This pathology not only has devastating health consequences but also entails considerable financial costs for individuals and communities [8]. To address this worldwide challenge, scientific research has focused on treating and preventing further increases in obesity rates. To this end, several research studies and pharmaceutical companies have concentrated on discovering and developing natural and synthetic drugs to reduce the damage caused by obesity.

Current pharmacological strategies for obesity management target central and peripheral pathways involved in appetite regulation and energy homeostasis. Early anti-obesity agents such as sibutramine and rimonabant were withdrawn due to severe cardiovascular and neuropsychiatric adverse effects, highlighting the limitations of centrally acting therapies [9,10,11]. More recently, glucagon-like peptide-1 receptor agonists, including semaglutide, liraglutide, and orforglipron have demonstrated robust efficacy in inducing weight loss by modulating appetite, gastric emptying, and insulin secretion [12,13,14]. In addition, these anti-obesity drugs can be administered orally or subcutaneously [15].

Despite their clinical success, GLP-1RA therapy is associated with a pattern of adverse events affecting their safety and tolerability [16], high cost [17], parenteral administration, long-term dependency, and gastrointestinal side effects, restricting their accessibility at the population level. Therefore, there is a growing interest in complementary or alternative approaches, particularly those derived from natural sources, to support safer and more sustainable obesity management strategies. Inhibition of human digestive gastric (HGL) and pancreatic (HPL) lipases to reduce fat absorption has become the main pharmacological approach for the treatment of obesity over the last decade [18,19]. The lipase inhibitor, Orlistat, marketed as Xenical^®^ or Alli^®^ exemplifies this approach. Orlistat, or tetrahydrolipstatin (THL), is a derivative of lipstatin that was isolated from *Streptomyces toxytricini* [20]. It is a potent anti-obesity drug that inhibits the hydrolysis of dietary triglycerides (TGs) into absorbable fatty acids and monoglycerides by inhibiting digestive lipases [21,22]. Orlistat has been reported to cause weight loss, inhibit fat absorption by 30%, and limit caloric intake [23]. In addition to its anti-obesity effect, Orlistat has been shown to have numerous undesirable effects, including liver damage [24]. Given the several side effects and the current prescribing guidelines for medications, it is crucial to discover new drugs with a high level of safety and efficacy.

For the management of obesity, and in addition to synthetic drugs, it has been reported that natural products, particularly extracts and isolated compounds, have been widely developed as anti-obesity agents in traditional medicine to treat and remediate obesity through different mechanisms, including the inhibition of digestive lipases. These products were used in the management of obesity and other pathologies due to various bioactive compounds, including terpenoids, flavonoids, and polyphenols. Many medicinal, herbal, and edible plants have shown potent anti-obesity effects through lipase inhibitory activity, such as *Phaseolus vulgaris* [25], *Invingia gabonensis* [26], *Chili pepper* [27] and some *Lauraceae* such as cinnamon [28,29]. However, although medicinal plants are often considered safer than synthetic drugs, they may contain toxic compounds that can compromise their safety [30]. Therefore, considerable efforts have been devoted to identifying natural lipase-inhibiting compounds and developing reliable approaches to isolate active molecules while reducing the potential adverse effects of crude extracts.

This study aimed to screen digestive lipase inhibitors from ethanolic extracts prepared from nine medicinal plants. The extract exhibiting the highest inhibitory activity was further fractionated to identify the compounds responsible for lipase inhibition. The most active fraction was analyzed by high-resolution tandem mass spectrometry (HRMS/MS). The identification of the putative active inhibitor and the proposed inhibition mechanism were investigated using proteomic approaches coupled with mass spectrometry, including peptide mass fingerprinting (PMF), together with molecular docking.

## 2. Results and Discussion

### 2.1. Screening for Lipase Inhibitor Properties of Medicinal Plants Crude Extract

During a primary screening, nine crude extracts, prepared from different parts of nine medicinal plants (Table S1) by extraction using ethanol, were tested for their inhibitory potential against PPL, initially chosen as the inhibition target. The inhibitory potential of all crude extracts was calculated as a percentage of the residual PPL activity after incubation with the target extract at a final concentration of 1 mg·mL^−1^. The results illustrated in Table 1 showed that the EE of *C. cassia* bark exhibited the highest inhibitory potential, with a residual PPL activity of 11 ± 2%. The EEs of *Ceratonia siliqua*, *Coffea arabica*, and *Linum usitatissimum* showed moderate inhibitor potential, with residual PPL activities of 38 ± 8%, 51 ± 9%, and 53 ± 7%, respectively (Table 1), whereas EEs from *Quercus macrocarpa*, *Cuminum cyminum*, *Zingiber officinale*, *Cucumis melo*, and *Thymus vulgaris* showed weak or negligible anti-lipase activity (residual activities > 60%) (Table 1). With outstanding inhibitory potential, *C. cassia* bark was selected for further bioactivity-guided fractionation and mechanistic studies. As previous studies have shown, *Cinnamomum* is a commercially available spice used in culinary and medicinal applications, cosmetics, and perfumery. Regarding medicinal applications, *Cinnamomum* has been used in both traditional and modern medicine to treat headaches, diabetes, digestive disorders, and obesity [31,32,33].

### 2.2. Evaluation of the Anti-Lipase Activity of Different Extracts of C. cassia

**Figure 1:**
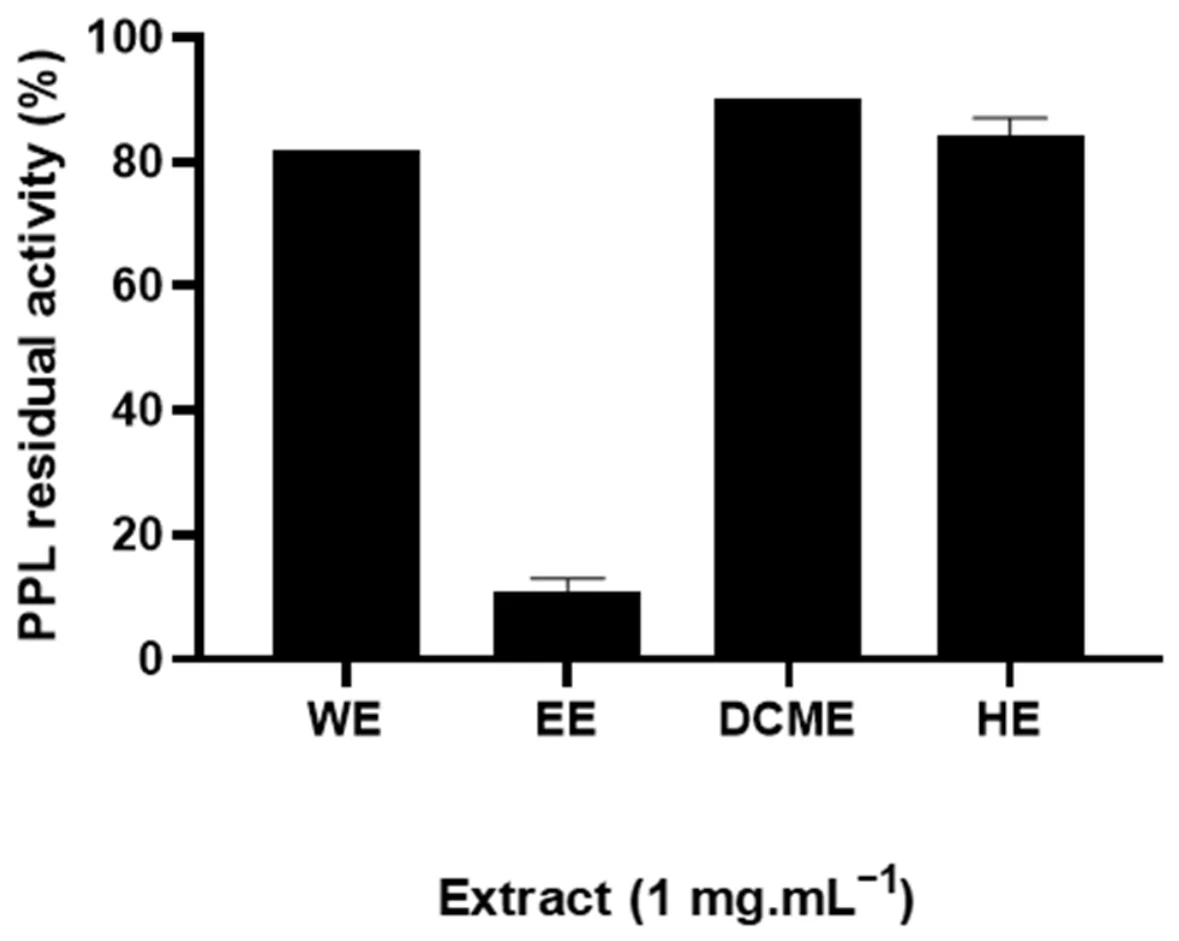
Inhibitory activities of four extracts of *C. cassia* against PPL using TC4 as substrate. HE, DCME, EE, and WE were tested at a final concentration of 1 mg mL^−1^. PPL (5 µg) was pre-incubated in the presence of HE, DCME, EE, or WE for 30 min at 25 °C, and the RA of PPL was measured. The percentage of inhibition (or RA (%)) was determined directly by comparing the steady-state reaction velocity or total product release (free fatty acids measured continuously by the pH-Stat technique) in the presence of the extract against a control without inhibitor (set to 100%). Data represent mean ± SD (n = 3).

The inhibitory potential of the HE, DCME, EE, and WE of *C. cassia* bark was determined. Using a final concentration of 1 mg·mL^−1^ of each of these extracts, the residual activity of PPL was shown to be 84 ± 3%, 90%, 11 ± 2%, and 82%, respectively (Figure 1). These results clearly demonstrate that the inhibitory compounds were extracted by ethanol. It has been shown previously that at a concentration of 200 µg·mL^−1^, the EE of *C. cassia* had a stronger inhibitory activity compared with the DCM: methanol extract, with PPL inhibitory activity of 80 ± 10% and 30 ± 6%, respectively [34]. However, it has been demonstrated that the HE of *Cinnamomum* did not display any inhibitory potential against turkey pancreatic lipase, with an RA of 100% [35]. In addition, a modern pharmacological study showed that the EE of *C. cassia* bark had hypolipedemic and anti-diabetic effects [36].

### 2.3. Bioactivity-Guided Fractionation

To further refine the bioactivity-guided fractionation of the EE and based on the results of the inhibitory potential of the different *C. cassia* extracts, we evaluated the inhibitory activity of the initial *C. cassia* dried bark following a sequential extraction process: initial delipidation with hexane followed by extraction with ethanol. The results demonstrated a significant enhancement in inhibitory potency: the RA (%) of PPL dropped to 1 ± 2% with the delipidated extract, compared to 11 ± 2% for the crude EE (Figure S1A). This marked increase in efficacy can be attributed to the delipidation step, which likely concentrated the primary inhibitory compounds while simultaneously removing antagonistic lipophilic components that may have interfered with the inhibitory effect. The defatted EE was analyzed by NP-TLC. A mobile phase of ethyl acetate/petroleum ether (6/4 *v*:*v*) provided the most effective separation (Figure S1B). The chromatogram shows at least six compounds with decreasing polarity from the baseline to the front of the solvent.

Based on these results, the defatted EE was fractionated using silica gel chromatography following the optimized protocol, yielding three distinct fractions: F1, F2, and F3 (Figure S2). Subsequent anti-lipase activity assay (Figure S2A) and NP-TLC analysis (Figure S2B) revealed that the original extract separated into three groups of compounds based on their polarity. The inhibitory assays showed that at a concentration of 0.5 µg·mL^−1^, the RA (%) activity of PPL was 80% ± 2, 49% ± 3, and 9% ± 5 for F1, F2, and F3, respectively (Figure S2A). Fraction F3 exhibited the highest inhibitory potency against PPL. NP-TLC analysis demonstrated that F3 possessed a highly polar character. Consequently, a second fractionation step was performed on a reversed-phase C18 column to further isolate the active compounds. Chromatographic separation of F3 yielded five distinct sub-fractions, designated F3-1 to F3-5 (Figure S3A). These were subsequently evaluated for their inhibitory activity against PPL and characterized by TLC. The results indicated that at a concentration of 100 µg·mL^−1^, the fraction F3-1 exhibited the most potent inhibitory potential with RA (%) of 18% ± 4. NP-TLC analysis revealed that fractions F3-1 and F3-2 exhibited minimal mobility, remaining largely retained by the stationary phase (Figure S3B). This behavior confirms the highly polar nature of these two fractions. Their polarity was further validated using RP-TLC with a highly polar mobile phase. Under these conditions, fraction F3-1 displayed a distinct, migrating spot, suggesting the presence of a specific inhibitory compound (Figure S3C). Based on these analytical results, we focused the subsequent characterization efforts on fraction F3-1 due to its relatively lower complexity.

To further isolate and enrich the active principle, fraction F3-1 was subjected to reversed-phase C18 column chromatography, and two distinct subfractions were eluted, F3-1-1 and F3-1-2 (Figure S4A). At a concentration of 100 µg·mL^−1^, the RA (%) of PPL was shown to be 3% ± 2 for F3-1-1 and 98% ± 4 for F3-1-2 (Figure S4B). Notably, the specific inhibitory potency increased significantly throughout the purification process; the amount of fraction required to inhibit PPL decreased tenfold after three successive fractionation steps. Before bioactivity-guided fractionation, the RA (%) of PPL was 9% ± 2 in the presence of 1 mg·mL^−1^ of the crude EE (Figure S2A), while at the end of this process, the RA (%) of PPL, measured under the same conditions, was 3% ± 2 in the presence of 100 µg·mL^−1^ of the enriched fraction F3-1-1 (Figure S4B). One of the hallmarks of bioactivity-guided fractionation is the trend of increasing potency with fractionation. Furthermore, previous reports suggest that this phyto-fractionation process holds promise for an efficient natural drug discovery approach [37]. Based on these results, fraction F3-1-1 was selected as the most potent lead to evaluate its inhibitory spectrum against other key digestive enzymes, specifically rHPL and rDGL.

### 2.4. The Inhibitory Potential of Fraction F3-1-1 Against Digestive Lipases

**Figure 2:**
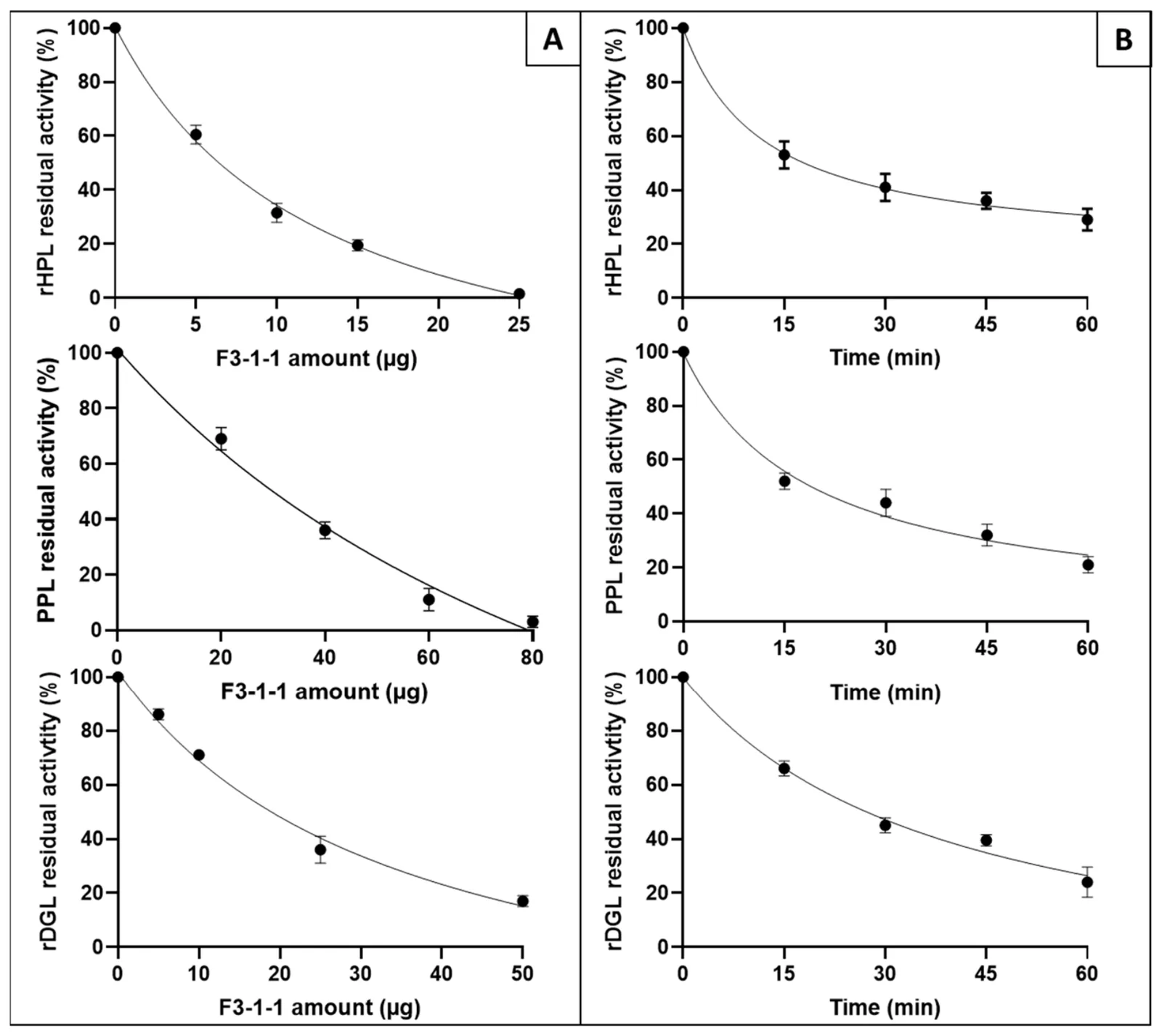
Evaluation of the inhibitory effect of fraction F3-1-1 against digestive lipases: rDGL, rHPL, and PPL. (**A**) Dose-dependent inhibition. Each lipase was pre-incubated with various amounts of F3-1-1 for 30 min at 25 °C in the presence of 4 mM NaTDC. RA (%) was measured at 37 °C using TC4 as substrate. (**B**) Time-dependent inhibition. Each lipase was pre-incubated with a constant amount of F3-1-1 (rHPL: 3 µg; PPL: 35 µg; rDGL: 20 µg) at 25 °C for increasing time periods (0 to 60 min). RA (%) was then measured as above. Data are means ± SD (n = 3).

The current study aims to identify natural-derived digestive lipase inhibitors in order to reduce lipid absorption for the treatment of obesity. We therefore evaluated the inhibitory potential of an enriched fraction of *C. cassia* against digestive lipases. Initially, the inhibitory activity of the crude extract and different fractions of *C. cassia* was assessed against PPL, which was used as the initial target for inhibitor screening. Subsequently, the inhibitory activity of fraction F3-1-1 was tested against purified rHPL and rDGL. PPL and rHPL share 85% identity [38], and rDGL is an interesting model enzyme for human gastric lipase (HGL). Indeed, the amino acid sequence identity between these two lipases is 85.7%, and their three-dimensional structures are superimposable [39]. The influence of the amount values of fraction F3-1-1 and pre-incubation times was evaluated using the enzyme and inhibitor pre-incubation method in the absence of the substrate, as indicated in the Section 3. The inhibition profiles thus obtained revealed that this fraction possesses a dose-dependent inhibitory activity (Figure 2A). RA (%) of 1% ± 2, 11% ± 4%, or 15% ± 2% were obtained after a 30 min pre-incubation of rHPL, PPL, or rDGL (0.5 µM, final concentration) with 25 µg, 60 µg, or 50 µg of the F3-1-1 fraction, respectively. The results obtained indicate that F3-1-1 inhibits rHPL much more effectively than PPL and rDGL, with α_I50_ values of 7 µg, 32 µg, and 19.5 µg, respectively (Table 2). Furthermore, rHPL is completely inhibited at an α_I_ of 25 µg after 30 min of pre-incubation, whereas PPL and rDGL retain 40% and 30% of their activity at an α_I_ of 40 µg (Figure 2A).

The influence of the pre-incubation time of rHPL, PPL, or rDGL with F3-1-1 was further evaluated (Figure 2B). In all cases, the residual activity of these lipases decreased rapidly, reaching approximately 40% after 30 min of pre-incubation. This residual activity then decreased slightly, reaching 35% for rHPL, 25% for PPL, and 28% for DGL after 1 h of pre-incubation in the presence of 8 µg, 35 µg, and 20 µg of F3-1-1, respectively (Figure 2B). From these inhibition graphs, the half-lives were then determined and found to be 17 min, 16 min, and 25 min for rHPL, PPL, and rDGL, respectively (Table 2). Such values of α_I50_ and t½ reflect strong inhibition of these lipases by F3-1-1.

Based on α_I50_ and t½ values, the F3-1-1 had the ability to inhibit the catalytic activity of digestive lipases, which are the main therapeutic target for obesity [19]. During the digestion phase of a test meal, HGL and HPL hydrolyze, respectively, 17.5% and 48.5% of the acyl chains of TGs, thus leading to the 66% hydrolysis necessary for the complete absorption of lipids from this same test meal [40,41]. Consequently, specific inhibition of both gastric and pancreatic lipases impairs gastrointestinal digestion, significantly reduces intestinal absorption of lipolysis products from ingested lipids, and thus decreases caloric intake [42]. Gastric and pancreatic lipases are therefore a promising therapeutic target for the treatment of obesity.

**Figure 3:**
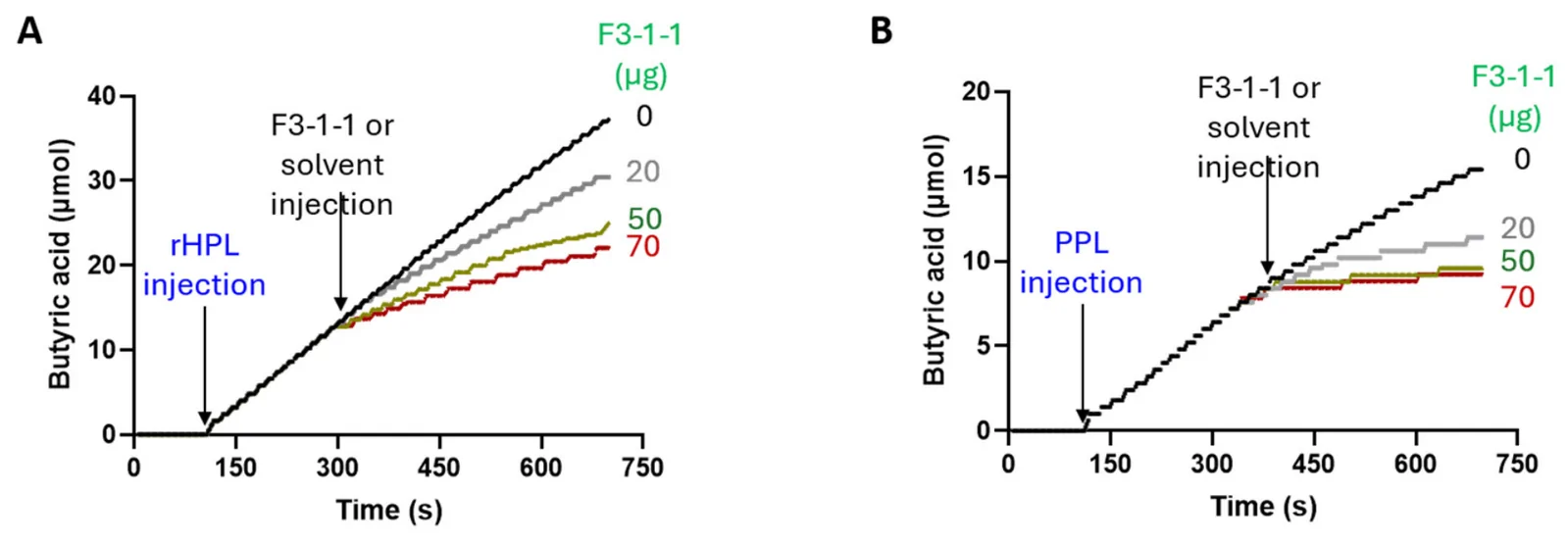
Effect of fraction F3-1-1 on the rate of TC4 hydrolysis by rHPL (**A**) and PPL (**B**) during ongoing lipolysis. After addition of the enzyme (0.5 µM, final concentration) to the reaction medium, fraction F3-1-1 at variable amounts (10–70 µg) was injected 4 min later (arrow). Assays were performed at 37 °C in a thermostated vessel containing 0.2 mL TC4, 2.5 mM Tris-HCl buffer (pH 8), 2 mM CaCl_2_, and 0.5 mM NaTDC. The rate of fatty acid release was monitored continuously by pH-stat. Each kinetic trace is representative of at least three independent experiments.

The influence of F3-1-1 on the rate of TC4 hydrolysis was evaluated for rHPL and PPL to test the possibility of inhibition of lipolytic activity during lipolysis. Four minutes after the addition of the enzyme, either solvent alone (ethanol/water 7/3, *v*:*v*) or F3-1-1 at variable amounts was injected. As shown from the kinetic curves in Figure 3, the rate of TC4 hydrolysis by the action of rHPL (Figure 3A) or PPL (Figure 3B) decreased after the addition of the inhibitor, but to different extents. The decrease in enzyme activity was more pronounced at higher F3-1-1 amounts. At a concentration of 70 µg of F3-1-1, approximately 36% and 12% inhibition of the enzyme activity of rHPL and PPL, respectively, was observed.

It is worth noting that inhibition of rHPL is very strong when the enzyme is pre-incubated with F3-1-1 in the presence of NaTDC compared to the method of injecting the inhibitor during lipolysis. This result shows that the inhibitor is primarily associated with the lipid phase, even in the presence of bile salts. This physicochemical property is probably a necessary condition for the effectiveness of the lipase inhibitor under physiological conditions. In the case of PPL, inhibition by F3-1-1 was more pronounced when the inhibitor was added during lipolysis than when the enzyme and inhibitor were pre-incubated in the absence of the substrate. This difference could be due either to the presence of other proteins in the pancreatic extract containing PPL, which exert a protective effect in the aqueous phase, or to the specific physicochemical properties of the inhibitor, or to the presence of a micellar concentration of NaTDC in the pre-incubation medium. Moreover, using olive oil as the substrate, the F3-1-1 against PPL was 52 µg (Figure S5), while it was 37 µg using TC4. Previous studies on lipase inhibitors showed that the rate of inhibition of dog pancreatic lipase and HPL differed depending on the method and substrate used, respectively [43,44].

### 2.5. UPLC-HRMS/MS-Based Chemical Characterization of Fraction F3-1-1

To characterize the main compounds present in fraction F3-1-1, UPLC-HRMS/MS analysis was performed. Figure S6 shows the total ion chromatogram profile of this fraction. Five major compounds were tentatively annotated based on their measured mass-to-charge ratios (*m*/*z*), proposed molecular formulas, MS/MS fragmentation patterns, database searches, and comparison with literature data, including previously reported natural compounds from *C. cassia*. The corresponding MS/MS data are presented in Table S3.

Compound **1** (R_T_ = 0.8 min), which has the highest intensity, was annotated as levoglucosan (1,6-Anhydro-beta-D-glucopyranose), a hexose with *m*/*z* 163.06 (C_6_H_10_O_5_) according to HMDB, Mass Bank, and Food Database [45].

Compounds **2** (R_T_ = 4 min) and 4 (R_T_ = 7.4 min) showed peaks with [M + H]^+^ at *m*/*z* 155.03 (C_7_H_6_O_4_) and *m*/*z* 123.04 (C_7_H_6_O_2_), and were annotated as a 3,5-dihydroxybenzoic acid and benzoic acid, respectively [46,47,48].

Compound **3** (R_T_ = 5.8 min), exhibiting a [M + H]^+^ ion at *m*/*z* 545.25 and consistent with the molecular formula C_26_H_40_O_12_, was annotated as cinncassiol A-19 glucoside based on database searches and literature data related to *C. cassia* [49]. This molecule is a diterpenoid previously isolated and characterized from the bark of *C. cassia* [50]. Diterpenoids from *Lauraceae,* and more specifically from *C. cassia,* have been shown to possess numerous biological activities, particularly in the treatment of tumors and chronic inflammatory diseases, and represent a major area of research in pharmacology, organic chemistry, and biosynthesis [51,52].

Compound **5** (R_T_ = 8 min) exhibit a [M + H]^−^ at *m*/*z* 185.08 (C_9_H_12_O_4_), and its MS-MS fragmentation gave predominant ions at *m*/*z* 139.07, *m*/*z* 93.07 and *m*/*z* 121.06 which were found in MS-MS spectra of 3,4,5-trimethoxyphenol [53,54]. This compound was already found and characterized in the bark of *C. cassia* [55].

### 2.6. Peptide Mass Fingerprinting and Molecular Docking Analysis

**Figure 4:**
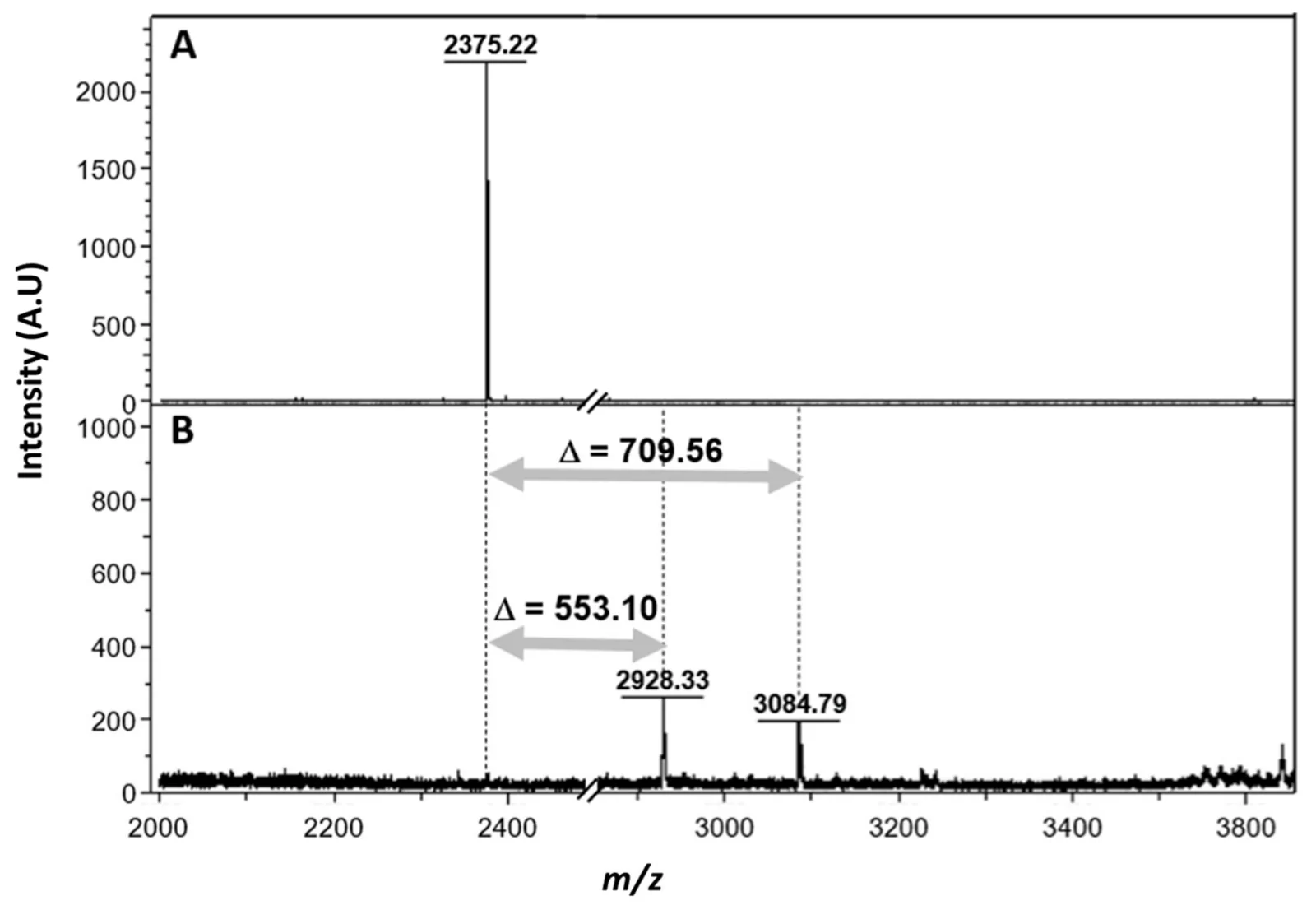
PMF spectra of untreated (**A**) and treated (**B**) rDGL with F3-1-1. A 30 min incubation of 2 µM rDGL was performed at 25 °C in the presence or absence of F3-1-1 (100 µg). The region of the unmodified isotopic catalytic peptide LHYVGHS^153^QGTTIGFIAFSTNPK containing the catalytic Ser153 was detected at [M − H]^+^ *m*/*z* 2375.22 Da (**A**). This peak disappears in the treated enzyme, where two peptides are detected at 2928.33 Da and 3084.78 Da (**B**), resulting from the covalent binding of the inhibitor of interest in F3-1-1 to the catalytic S^153^. Mass shift was calculated as the difference between the experimental *m*/*z* of the modified peptide and that of the unmodified catalytic peptide.

A proteomic approach combined with MALDI-TOF mass spectrometry was used to identify the inhibitor in the F3-1-1 fraction and to investigate the nature of the inhibition mechanism. After trypsin digestion, peptides from untreated and F3-1-1-treated rDGL were analyzed by mass spectrometry. For the untreated enzyme, the catalytic peptide L147-K168 containing the catalytic S153 (LHYVGH**S^153^**QGTTIGFIAFSTNPK, theoretical mass 2374.21 Da) was detected at [M + H]^+^ = 2375.25 Da (Figure 4A). In contrast, for rDGL pre-incubated with F3-1-1, no mass signal was detected at this *m*/*z* ratio, and two new mass signals were detected at *m*/*z* = 2928.33 Da and *m*/*z* = 3084.78 Da (Figure 4B). In the presence of F3-1-1, an increase in the mass of the catalytic peptide and two mass increments of +553.10 Da and +709.56 Da were observed compared to the untreated enzyme (Figure 4B). These mass variations between F3-1-1-treated and untreated rDGL could be due to specific coupling between catalytic S^153^ and a component of F3-1-1. Based on the UPLC-HRMS and MS/MS results of this fraction shown previously, the mass increment of +553.10 could be due to a covalent attachment of cinncassiol-related species detected in F3-1-1. Furthermore, it is worth noting that the F3-1-1-treated enzyme was completely inactivated. The second mass shift corresponding to +709.56 Da, observed in the enzyme treated with F3-1-1, could result from the fixation of cinncassiol A-19 itself linked to a levoglucosan residue previously detected in the fraction (544.25 Da + 163.06 Da = 707.31 Da).

These results, along with the observed mass shift in the treated enzyme, could be due to a strong bond with high energy of the DGL-inhibitor, likely involving the catalytic Ser^153^ of the enzyme with cinncassiol A-19. Other similar interactions between lipase and the inhibitor have been reported in the literature. MALDI-TOF results have previously shown an increase of approximately 500 Da in the molecular mass of HPL, and this increase most likely reflects the covalent bonding of the S^152^ of the HPL active site and the β-lactone ring of Orlistat (MW = 496 Da) [44]. Similarly, an enriched extract of star anise revealed the presence of a covalent inhibitor–enzyme complex by MALDI-TOF, with a mass increment of 489.52 Da. This specific coupling is probably due to the binding of myricitrin-5-methyl ether (479.11 Da) with rDGL [56].

**Figure 5:**
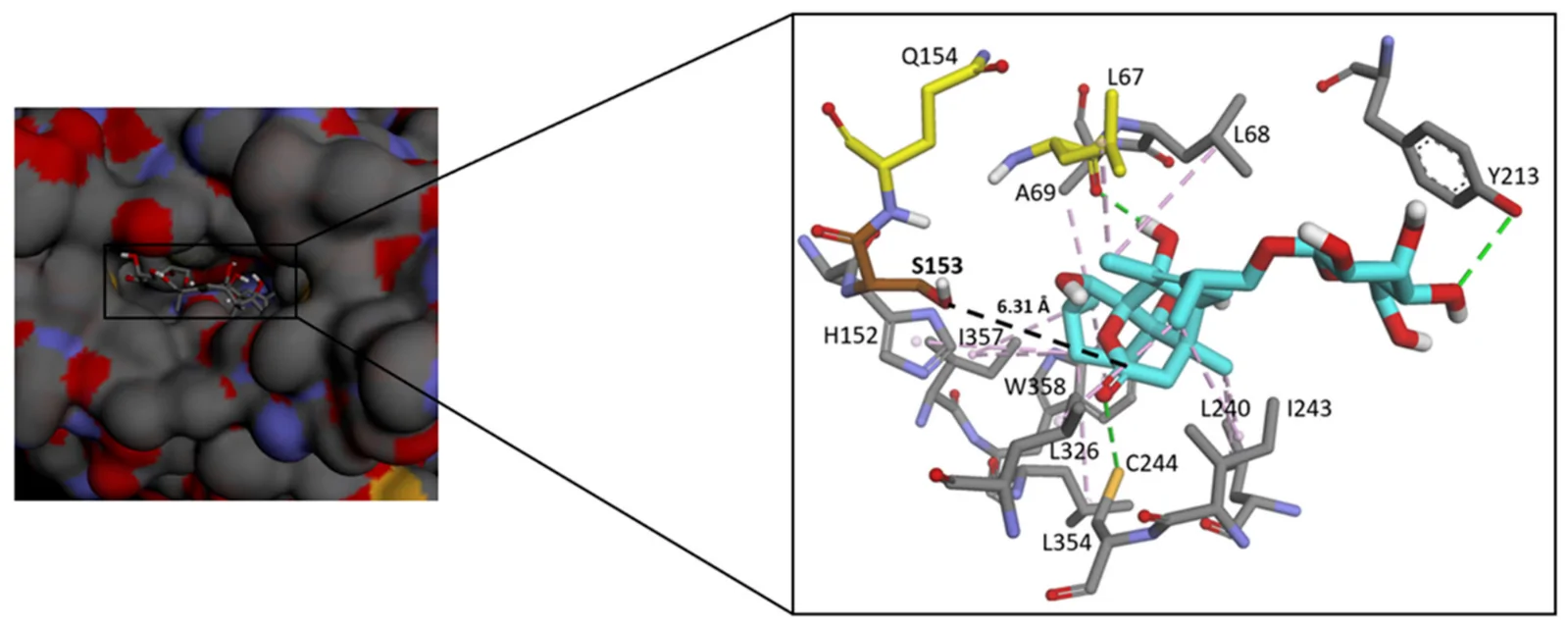
Visualization of the HGL-cinncassiol A-19 glucoside binding interaction by molecular docking. Molecular surface representation of the HGL active site crevice with the bound cinncassiol A-19 glucoside, stabilized by hydrogen bonding and hydrophobic contacts. O(seryl)-C(carbonyl) bond is shown as a black dotted line. Residues of the oxyanion hole (L67, Q254) are shown in yellow, and the catalytic serine residue (S153) is shown in brown. Hydrophobic interactions are shown as pink dotted lines, and hydrogen bonds are shown as green dotted lines.

To investigate HGL-cinncassiol A 19 glucoside binding interactions, in silico molecular docking was performed using VINA software(Autodock Vina 1.2.7) (Figure 5). The open conformation of HGL, in complex with THL covalently bound to its catalytic residue Ser^153^, was used as the reference model to improve the reliability of covalent binding. The active site of HGL was found to be accessible, thus enabling docking experiments. Automated docking resulted in several possible conformations of Cinncassiol A 19 glycoside within the HGL catalytic pocket, with a favorable binding energy of −7.6 kcal mol^−1^. The binding affinity of the HGL-Cinncassiol A 19 glycoside complex was maintained with three hydrogen bonds and nine hydrophobic interactions. For the docking results of the complex THL-HGL, a favorable binding energy of −7.9 kcal mol^−1^ was found. For subsequent analysis, we retained the best-matching conformation that directly exposes the lactone carbonyl residue of the Cinncassiol A 19 glycoside inhibitor towards the oxygen group of Ser^153^, within a calculated distance of 6.31 Å, which is sufficient to promote the nucleophilic attack. In the case of THL, the distance was found to be around 2.02 Å, which explains the strong inhibitory effect of THL on HGL.

**Figure 6:**
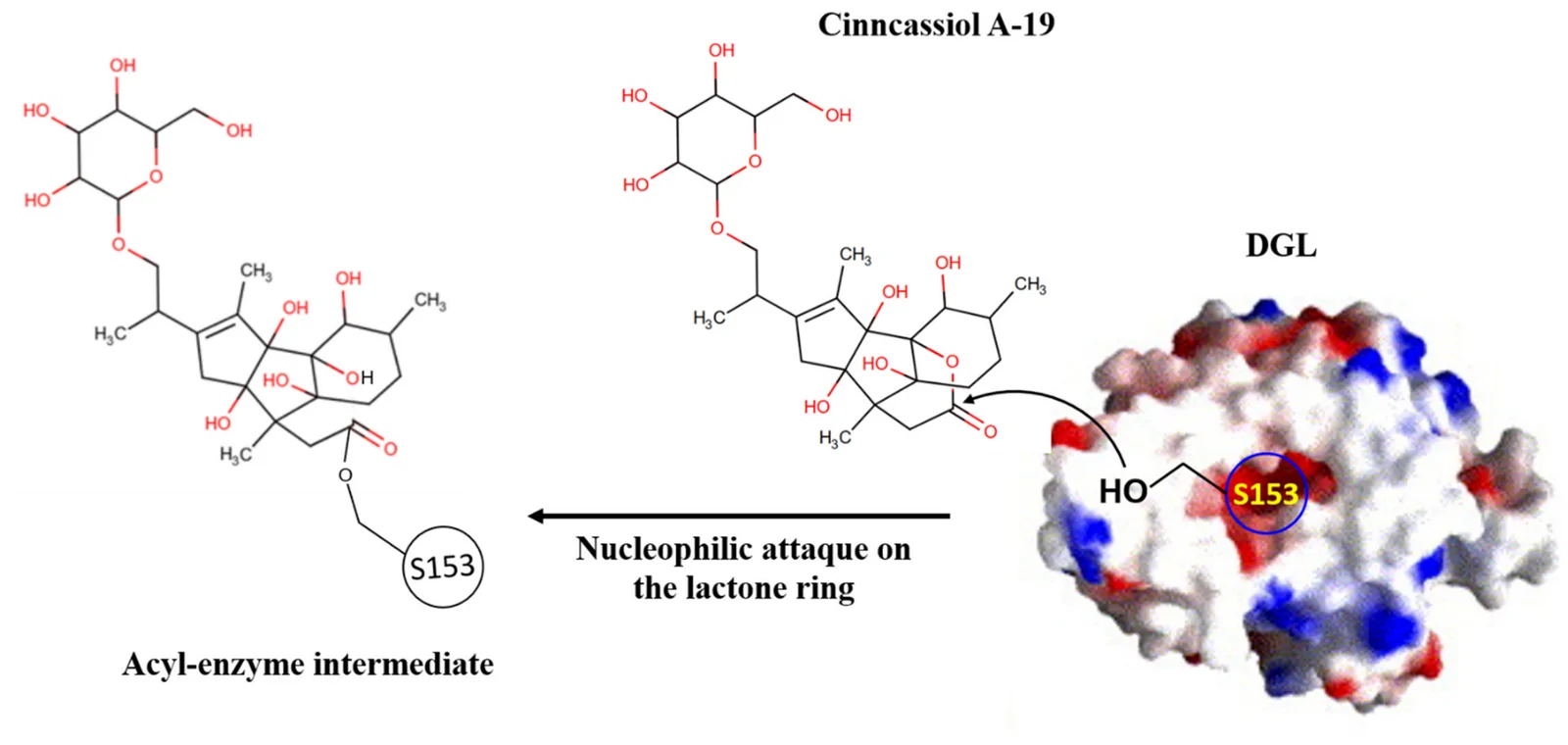
Proposed mechanism of covalent inhibition of gastric lipase by cinncassiol A-19 glucoside. The hydroxyl group of the catalytic Ser153 performs a nucleophilic attack on the carbonyl carbon of the lactone ring present in cinncassiol A-19 glucoside. This leads to the formation of a stable acyl-enzyme covalent intermediate, which inactivates the enzyme. The same mechanism is proposed for the di-glycosylated form (cinncassiol A-19 diglucoside). R represents the glucosyl or diglucosyl moiety. This mechanism is analogous to that of Orlistat.

Indeed, cinncassiol A-19 glucoside is a diterpene containing a lactone group that can be the target of nucleophilic attack by the catalytic S^153^ of the enzyme (Figure 6).

## 3. Material and Methods

### 3.1. Chemicals and Reagents

Sodium taurodeoxycholate (NaTDC), Glyceryl tributyrate (TC4), olive oil, bovine serum albumin (BSA) and gum Arabic (GA) were obtained from Sigma-Aldrich-Fluka (Saint-Quentin-Fallavier, France). Orlistat was purchased from Hoffmann-La-Roche Ltd. in Basel, Switzerland. Ethanol absolute anhydrous, methanol, petroleum ether, hexane, diethyl ether, dichloromethane (DCM) and acetonitrile (HPLC gradient grade) were obtained from Carlo Erba reagents (Val de Reuil, France). Normal-phase thin-layer chromatography (NP-TLC) aluminum sheets pre-coated with silica gel 60 and reversed-phase (RP)-18 TLC plates were obtained from Merck (Darmstadt, Germany). All other chemicals and solvents, of the highest quality, were obtained from local suppliers. Recombinant dog gastric lipase (rDGL, molecular mass, 49 kDa) was produced and purified as described previously [39]. rDGL is an interesting model enzyme for human gastric lipase (HGL). Indeed, the amino acid sequence identity between these two lipases is 85.7%, and their three-dimensional structures are superimposable [39]. Recombinant human pancreatic lipase (rHPL, molecular mass, 50 kDa) was expressed and purified in the laboratory. Porcine pancreatic lipase (PPL, Type II, L3126, molecular mass, 50 kDa) was purchased from Sigma-Aldrich-Fluka Chimie (Saint-Quentin-Fallavier, France). In addition, PPL and rHPL share 85% identity [38]. Pancreatic colipase was purified from lipid-free porcine pancreatic powder as described previously [57]. In all the experiments, water was obtained from the Milli-Q Gradient water purification system.

### 3.2. Preparation of Plant Crude Extracts

Nine dried plants from different regions of Tunisia (Table S1) were chosen based on traditional uses and ethnopharmacological relevance, with existing literature supporting their anti-obesity effects: *C. cassia* [58], *Ceratonia siliqua* [59], *Coffea arabica* [60], *Linum usitatissimum* [61], *Quercus* [62], *Cuminum cyminum* [63], *Zingiber officinale* [64], *Cucumis melo* [65], and *Thymus vulgaris* [66]. Dried plant parts were finely ground using a high-speed laboratory blender and then sieved to obtain powder. Each dried powder was extracted by maceration for 24 h with ethanol (1:10, *w*/*v*). The different extracts were filtered through No. 3 sintered glass and concentrated using a vacuum rotary evaporator. The dried powders were stored at 4 °C until analysis.

Prior to biological assays, for each extract, a stock solution with a final concentration of 100 mg·mL^−1^ was prepared in ethanol prior to the experiments. These extracts were then tested in vitro for their inhibitory activity against PPL, which was used as the initial inhibition target for the initial screening of digestive lipase inhibitors.

### 3.3. Preparation of the Different Cinnamomum cassia Extracts

Dried bark *C. cassia* powder was extracted, separately, by maceration with each of the following solvents (1:10, *w*/*v*): hexane, DCM, ethanol and water. The different extracts were filtered through No. 3 sintered glass and concentrated using a vacuum rotary evaporator. In the case of the aqueous extract, it was lyophilized. The inhibitory potential of Hexane extract (HE), DCM extract (DCME), Ethanol Extract (EE), and Water Extract (WE) was then tested against PPL.

### 3.4. Lipolytic Activity Measurement

Lipolytic activity was measured at 37 °C by automated, continuous titration of fatty acids released, under the catalytic action of lipase on a mechanically stirred TG emulsion, using 0.1 M NaOH and a pH-STAT (Metrohm 702 SM Titrino, Herisau, Switzerland) calibrated to a fixed end point. For PPL and rHPL, the enzymatic assay was performed in a thermostated vessel (37 °C) containing 0.2 mL of TC4 and 9.8 mL of 2.5 mM Tris-HCl buffer, 150 mM NaCl buffer (pH 8), 2 mM CaCl_2_, and 0.5 mM NaTDC.

When olive oil was used as the substrate, it was first pre-emulsified with GA, as previously described [67]. Five mL of this emulsion was added to 10 mL of 2.5 mM Tris-HCl, 150 mM NaCl buffer (pH 8) containing 2 mM CaCl_2_ and 0.5 mM NaTDC. When required, colipase was added in a molar excess of 5 relative to rHPL in the presence of 4 mM NaTDC.

In the case of rDGL activity measurement, the enzyme was assayed at pH 5.5 using TC4 as substrate and the standard assay solution: 150 mM NaCl, 2 mM NaTDC, and 2 µM BSA [39]. Enzyme activity was expressed in international units (U), where one unit corresponds to the release of 1 µmol of fatty acid per minute under the assay conditions. For all experiments, the lipase activities were tested at a concentration of 0.5 µM.

### 3.5. Methods to Test Lipase Inhibition

The inhibition of lipase activity by the medicinal plant extract was determined using two methods:

- Lipase inhibitor pre-incubation method: It involves pre-incubating the lipase with the inhibitor in the absence of a substrate. This method was designed to visualize, in an aqueous medium, the potential direct interaction between the lipase and the inhibitor [43,44]. Pre-incubations of the lipase with various inhibitor amounts were carried out at room temperature in the presence of NaTDC (4 mM, final concentration) in order to create a micellar interface that may contain the inhibitor [44,68].
- Lipase inhibition during lipolysis method: This method involves adding the inhibitor a few minutes after injecting the lipase into the reaction medium containing the emulsified substrate. This method was designed to test for potential inhibition of the lipase-catalyzed hydrolysis reaction during lipolysis [44].

The degree of inhibition was assessed by measuring the relative residual lipase activity (RA%), which is the ratio of lipase activity in the presence and absence of the inhibitor.

$$\mathrm{RA}\;\%=\frac{Enzymatic\;activity\;in\;the\;presence\;of\;the\;inhibitor}{Enzymatic\;activity\;in\;the\;absence\;of\;the\;inhibitor\;}\;*\;100$$

The amount of extract required to achieve 50% inhibition (α_I50_) was determined, with α_I50_ denoting the mass (expressed in micrograms) of the extract or fraction needed to reduce the activity by half. The half-inactivation time (t½) was defined as the time required to reach 50% of the residual lipase activity for a given value of extract amount (α_I_). A control sample, for each experiment, was performed in the absence of the inhibitor fraction but with the same volume of solvent in which the inhibitor was solubilized (ethanol: water, 7:3 *v*/*v*).

### 3.6. Bioactivity-Guided Fractionation

**Figure 7:**
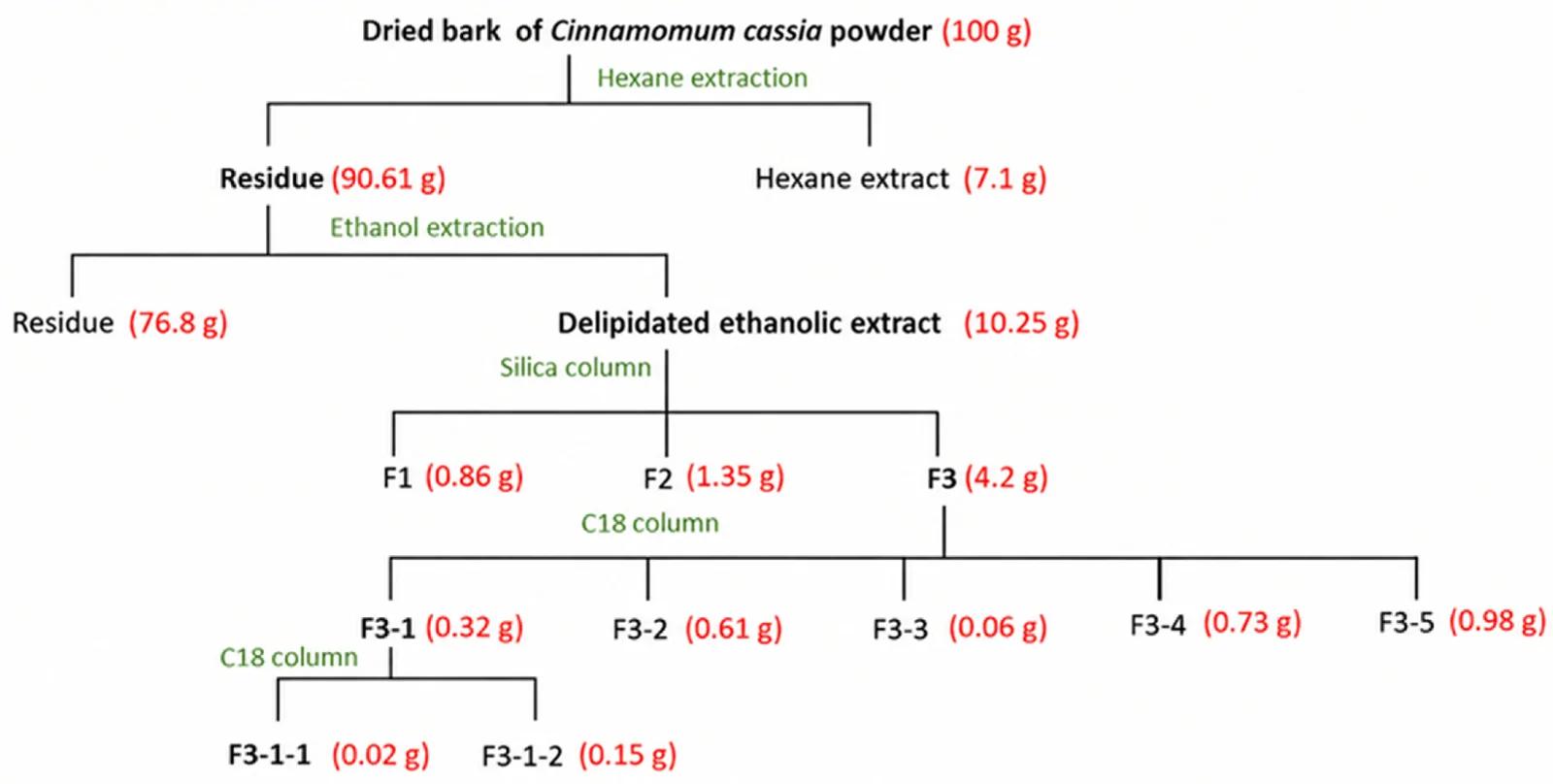
Bioactivity-guided fractionation scheme of the crude ethanolic extract of *Cinnamomum cassia* bark. The workflow illustrates the sequential extraction, defatting, silica gel column chromatography, reversed-phase C18 column chromatography steps, and the corresponding inhibitory activity against PPL measured at each stage. The most active fraction (F3-1-1) is highlighted.

This part of the work was carried out in five steps. The first two steps consisted of two successive macerations to concentrate the potentially inhibitory compounds. After each phytochemical step, the inhibition activity against PPL was checked. In the first maceration, 100 g of dried bark of *C. cassia* powder was macerated in hexane (1:10, *w*:*v*). After 24 h, the mixture was filtered using a sintered glass funnel; the liquid phase was evaporated to dryness under vacuum, and the solid residue was dried under a fume hood. In the second maceration, this defatted residue was extracted with ethanol for 24 h. The delipidated EE was filtered on a sintered glass funnel, and the resulting filtered crude extract was vacuum-dried and analyzed by NP-TLC using various solvent systems (water, DCM, methanol, ethyl acetate, and petroleum ether) in different ratios. In the third step, the delipidated EE was fractionated using silica gel column chromatography into three fractions (F1, F2, F3), using three different mobile phases: ethyl acetate/petroleum ether (5:5, *v*:*v*) (F1), ethyl acetate (F2), and methanol (F3). The resulting fractions were evaporated similarly and analyzed by TLC. Migration was performed with an ethyl acetate: cyclohexane (3:7, *v*/*v*) for the NP-TLC, or water: acetonitrile (0.1:1, *v*:*v*) for the RP-TLC. The spots were revealed by UV at 320 nm. The inhibitory potential was tested against PPL. In the fourth step, based on the inhibitory potential of these fractions, the methanolic fraction (F3) was fractionated into five subfractions (F3-1 to F3-5) by liquid chromatography (LC) on a C18 column, using acetonitrile and water as the mobile phase following an optimized gradient (Table S2). A RP-TLC analysis was then performed using migration solvent water: acetonitrile (0.1:1, *v*:*v*). Finally, based on the TLC results, fraction F3-1 was further separated by LC on the C18 column using solvent gradients shown in Supplementary Data. Figure 7 illustrates the different fractions of *C. cassia* powder.

### 3.7. UPLC-HRMS/MS Analysis of Active Fractions

The analysis of *C. Cassia* fractions was performed using an Ultimate 3000 ultra-performance liquid chromatography (UPLC) (Ultimate 3000, Thermo, Fisher Scientific, Waltham, MA, USA) system coupled to a high-resolution hybrid quadrupole time-of-flight (Q-TOF) mass spectrometer (Impact II Q-TOF, Bruker, Billerica, MA, USA). Chromatographic separation was carried out on a Luna Omega C18 column (100 × 2.1 mm, 1.6 µm, Phenomenex, Torrance, CA, USA). Samples were dissolved in methanol at a final concentration of 10 mg·mL^−1^. The column temperature was set at 40 °C, and the injection volume was 2 µL. Separation was performed at a flow rate of 400 µL min^−1^, using mobile phase A, water containing 0.1% formic acid, and mobile phase B, methanol/acetonitrile (50:50, *v*/*v*) containing 0.1% formic acid. The following linear gradient was applied: 100% A from 0 to 18 min, 100–0% A from 18 to 28 min, 0–100% A from 28 to 28.2 min, followed by column re-equilibration at 100% A until 35 min.

The mass spectrometry detection was performed in full-scan mode over the *m*/*z* range 20–1000, using both positive and negative electrospray ionization modes (ESI^+^, ESI^−^). The source temperature was set to 200 °C, the nebulizer gas pressure to 45 psi, and the capillary voltage to 4500 V in positive mode and 3500 V in negative mode. For MS/MS experiments, low-energy collision-induced dissociation (CID) was performed using collision energies ranging from 20 to 47 eV.

### 3.8. MS Data Analysis

Raw data obtained from UPLC-HRMS/MS analysis were converted to mzML and mgf formats using MSConvert software (ver. 3.0.2) and the Data Analysis module. The chromatogram was generated with a group size of several scans, a minimum group intensity of 1 × 10^4^, and a minimum absolute intensity of 5.0 × 10^5^, with an *m*/*z* tolerance of 5 ppm and mSigma < 30. The ion identity networking method was done with an *m*/*z* tolerance of 3.5. The adducts [M + H]^+^ and [M − H]^−^ were set for the positive and negative modes, respectively. Peaks processed from the sample were filtered based on peaks present in the blank sample (100% methanol), which was prepared under the same conditions as the sample, and by subtracting the blank. The remaining peaks were then analyzed. Molecular networking and library search were performed using the global natural products social (GNPS) molecular networking software (Phenix Version 1.21.2-5419) (https://gnps2.org), the Food Database, Mass Bank, and the human data base (HMDB). For the molecular networking, the peaks were uploaded to the GNPS platform, and the precursor ion Mass Tolerance and fragment ion Mass Tolerance were set at 0.02 Da and 0.05 Da, respectively. A cosine score threshold of 0.7 and a minimum of 6 matching fragment ions were required, each, to perform spectral searches and analysis against GNPS2 libraries.

### 3.9. Peptide Mass Fingerprinting

An aliquot of rDGL (12 µg) was pre-incubated with F-3-1-1 in the presence of 4 mM NaTDC. After 30 min, inhibition was tested using an anti-lipase activity assay. The mixture was then separated by 12% SDS-PAGE. A control was prepared under the same conditions, without F-3-1-1. Protein bands corresponding to 50 kDa were excised from the gel as previously described [69]. For protein identification and molecular mass determination, protein bands were digested overnight at 37 °C with a Trypsin/Lys-C protease mixture (Promega, Madison, WI, USA). The resulting tryptic peptide digest was analyzed by MALDI-TOF mass spectrometry using a Microflex II spectrometer (Bruker Daltonics, Bremen, Germany). For peptide mass analysis, spectra were acquired in positive reflectron mode over the 600–5000 Da mass range. External mass calibration was performed using the monoisotopic [M + H]^+^ ions of the following peptide calibration standards such as Angiotensin II (*m*/*z* 1046.5418), Angiotensin I (*m*/*z* 1296.6848), substance P (*m*/*z* 1347.7354), Bombesin (*m*/*z* 1619.8223), ACTH clip1-17 (*m*/*z* 2093.0862), ACTH clip18-39 (*m*/*z* 2465.1983), somatostatin 28 (*m*/*z* 3147.4710), as previously described in [70]. Briefly, tryptic digests were spotted directly onto a MALDI stainless-steel MALDI target plate. An equal volume of saturated α-cyano-4-hydroxycinnamic acid matrix solution prepared in acetonitrile/water/trifluoroacetic acid (70:29.9:0.1, *v*/*v*/*v*) was added to each spot, which was then allowed to dry at room temperature. Peak lists were generated using Flex Analysis software version 3.4 and manually inspected. Experimentally measured peptide masses were compared to the theoretical masses of tryptic peptides calculated from the rDGL amino acid sequence, including putative variable modifications of the catalytic serine residue corresponding to adduct formation with compounds present in fraction F3-1-1 [70].

### 3.10. Molecular Docking

In silico molecular docking of Cinncassiol A-19 glycoside and THL in the active site of HGL (PDB entry codes: 1HLG) was performed using VINA software. The grid box size was chosen to fit the whole active site cleft and to allow non-constructive binding positions. The inhibitor structure model was built using Phenix’s eLBOW version 2.2.1-6174 [71]. The most energetically favorable conformations were selected for further analysis. Protein–ligand interactions, including hydrogen bonding, hydrophobic contacts, and proximity to the catalytic triad, were analyzed to evaluate the potential inhibitory mechanism.

This computational approach was used to complement experimental findings and provide structural insights into the interaction between cinncassiol A-19 glucoside and gastric lipase, particularly in comparison with the well-characterized mechanism of covalent inhibitor THL interaction with lipase.

### 3.11. Statistical Analysis

Statistical analysis was performed using GraphPad Prism version 8.0.1. Concentration–response curves were fitted by nonlinear regression, and α_I50_ values were calculated accordingly. The 95% confidence intervals (95% CI) of the fitted parameters were estimated using the profile likelihood method. Data are presented as mean ± standard deviation (SD) from at least three independent experiments. Differences were considered statistically significant at *p* < 0.05.

## 4. Conclusions and Perspectives

Using a rigorous bioactivity-guided fractionation workflow, we isolated fraction F3-1-1, which potently inhibits rHPL, PPL, and rDGL, with α_I50_ values of 7 µg, 32 µg, and 19.5 µg, respectively. UPLC-HRMS/MS analysis annotated cinncassiol A-19 glucoside as a potential bioactive constituent. For the first time, PMF demonstrated that, in the presence of the enriched fraction F3-1-1, a compound covalently binds to the catalytic peptide containing Ser153 of gastric lipase, forming stable adducts characterized by mass shifts of +553.10 Da and +709.56 Da.

These mass shifts could be due to the binding of cinncassiol A-19 glucoside and cinncassiol A-19 glucoside associated with a levoglucosan residue, respectively. These findings are particularly significant from a lipase inhibitor perspective, but they need to be confirmed with a pure compound. However, cinncassiol A-19 glucoside is not available for purchase. Total synthesis of cinncassiol A-19 glucoside would be a significant advance to establish precise structure–activity relationships and define optimal dosing regimens.

## Figures and Tables

**Table 1:** Inhibitory effect of different medicinal plant extracts against PPL.

Plant	PPL Residual Activity (%)
*Cinnamonum cassia*	11 ± 2
*Ceratonia siliqua*	38 ± 8
*Quercus macrocarpa*	74 ± 5
*Coffea arabica*	51 ± 9
*Cuminum cyminum*	62 ± 13
*Zingiber officinale*	81 ± 1
*Linum usitatissimum*	53 ± 7
*Cucumis melo*	96 ± 5
*Thymus vulgaris*	86 ± 11

**Table 2:** α_I50_ and t½ values of F3-1-1 on rHPL, PPL and rDGL. t½ was determined by incubating 8 µg, 35 µg, and 20 µg of F3-1-1 with rHPL, PPL, and rDGL, respectively. Results were expressed as mean values of at least three independent experiments.

Enzyme	rHPL	PPL	rDGL
**α_I50_** (µg)	7	32	19.5
**t½** (min)	17	16	25

## Data Availability

The original contributions presented in this study are included in the article/Supplementary Material. Further inquiries can be directed to the corresponding author.

## Supplementary Materials

The following supporting information can be downloaded at: https://www.mdpi.com/article/10.3390/molecules31203737/s1.

## Abbreviations

BSA, bovine serum albumin; DCM, dichloromethane; DCME, dichloromethane extract; EE, ethanol extract; ESI, electrospray ionization; GA, gum Arabic; GNPS, global natural products social molecular networking; HE, hexane extract; HGL, human gastric lipase; HMDB, human metabolome database; HRMS, high-resolution mass spectrometry; MS/MS, tandem mass spectrometry; NaTDC, sodium taurodeoxycholate; NP-TLC, normal-phase thin-layer chromatography; PMF, peptide mass fingerprinting; PPL, porcine pancreatic lipase; Q-TOF, quadrupole time-of-flight; RA, residual activity; rDGL, recombinant dog gastric lipase; rHPL, recombinant HPL; RP-LC, reversed-phase liquid chromatography; RP-TLC, reversed-phase thin-layer chromatography; R_T_, retention time; TC4, glyceryl tributyrate; THL, tetrahydrolipstatin; UPLC, ultra-performance liquid chromatography; WE, water extract; α_I50_, amount of inhibitor required to reduce enzyme activity by 50%; t½, half-inactivation time.
