# Supplementary material for: Identification of an Inhibitory Fraction from Cinnamomum cassia Against Digestive Lipases
Source: Molecules. 2026 Oct 18;31(20):3737. doi: 10.3390/molecules31203737 (PMC13707961; doi:10.3390/molecules31203737)

SUPPLEMENTARY MATERIAL

Identification of an inhibitory fraction from *Cinnamomum cassia* against Digestive Lipases

Sihem Snini[1,2], Jannet Kamoun[2], Peter Balogun[1], Michele Fiore[1], Sébastien Violot[3], Mohamed Sellami[2], Sophie Ayciriex[1,4], Alexandre Noiriel[1], Ahmed Aloulou[2*], Abdelkarim Abousalham[1*]

[1] Université Lyon 1, CNRS, ICBMS, UMR 5246, Villeurbanne, France

[2] University of Sfax, National Engineering School of Sfax, Laboratory of Biochemistry and Enzymatic Engineering of Lipases, 3038 Sfax, Tunisia.

[3] Molecular Microbiology and Structural Biochemistry (MMSB), University of Lyon 1, UMR 5086 CNRS, 7 Passage du Vercors, Cedex 07, F-69367 Lyon, France.

[4] The Lyon Hepatology Institute, IHU EVEREST, Lyon, France

[*] Correspondence: abousalham@univ-lyon1.fr

Table S1. List of medicinal plant species, the plant part used, and the botanical family included in this study.

Plant	Part	Family
Cinnamomum cassia	Bark	*Lauraceae*
Ceratonia siliqua	Fruit	*Fabaceae*
Quercus macrocarpa	Fruit	*Fabaceae*
Coffea arabica	Grain	*Rubiaceae*
Cuminum cyminum	Grain	*Apiaceae*
Zingiber officinale	Fruit	*Zingiberaceae*
Linum usitatissimum	Grain	*Linaceae*
Cucumis melo	Grain	*Cucurbitaceae*
Thymus vulgaris	Whole	*Lamiaceae*

Table S2. UPLC gradient used for the purification of fraction F3 and subfraction F3-1 by reversed-phase C18 column chromatography. Mobile phase A: water, mobile phase B: acetonitrile.

**F3 separation gradients**		
Time (minutes)	A (%)	B (%)
0	90	10
8	90	10
13	70	30
20.5	70	30
27	10	90
30	10	90
39.5	0	100
45.5	0	100
**F3-1 separation gradients**		
Time (minutes)	A (%)	B (%)
0	80	20
5	70	30
10	70	30
13	10	90
18	10	90
18.2	0	100
22	0	100

Table S3. Annotation of compounds detected in fraction F3-1-1 by UPLC-HRMS and MS/MS fragmentation data: retention time (R_T), molecular formula, observed $[M+H]^+$ or $[M-H]^-$ *m/z*, mass error (ppm), and MS/MS fragment ions.

m/z	z	Adduct	R_T (min)	Empirical formula	Error (ppm)	Isotopic pattern (mSigma)	Compound
163.06	+1	M+H	0.8	$C_6H_{10}O_5$	-0.2	0.6	1,6-Anhydro-β-D-glucopyranose
155.03	+1	M+H	4.0	$C_7H_6O_4$	-1.3	4.8	3, 5 dihydroxybenzoic acid
545.25	+1	M+H	5.8	$C_{26}H_{40}O_{12}$	0.7	1.8	Cinncassiol A- 19 glucoside
123.04	+1	M+H	7.4	$C_7H_6O_2$	-0.3	6.5	Benzoic acid
185.08	+1	M+H	8.0	$C_9H_{12}O_5$	-1.5	2.5	3,4,5-trimethoxyphenol

Figure S1. (**A**) Inhibitory activities of crude EE and delipidated EE of *C. cassia* against PPL at a final concentration of 1 mg mL^{-1}. Defatting significantly enhanced inhibitory potency (RA (%): 1 ± 2% vs. 11 ± 2% for crude EE). (**B**) NP-TLC analysis of the delipidated EE revealed under UV light at 366 nm. Lanes 1–3: decreasing amounts of delipidated EE. Solvent system: ethyl acetate/petroleum ether (6:4, v/v). At least six compounds of decreasing polarity are visible.

Figure S2. (**A**) Inhibitory activities of fractions F1, F2, and F3 (eluted from silica gel column) against PPL using TC4 as substrate. Fractions were tested at 0.5 mg mL^{-1}. F3 showed the highest inhibition (residual activity: 9 ± 5%). (**B**) NP-TLC analysis of fractions F1, F2, and F3. Solvent system: ethyl acetate/cyclohexane (6:4, v/v). Revelation under UV-366 nm. F3 is highly polar (remains near the baseline).

Figure S3. (**A**) Chromatogram of fraction F3 purification using a C18 reversed-phase column, yielding five subfractions: F3-1(a), F3-2 (b), F3-3 (c), F3-4 (d), F3-5 (e). cv, column volume. (**B**) NP-TLC analysis of subfractions F3-1 to F3-5. Solvent system: ethyl acetate/cyclohexane (6:4, v/v). (C) RP-TLC analysis of subfractions F3-1 to F3-5. Solvent system: acetonitrile/water (0.1:1, v/v). Chromatograms revealed under UV-366 nm. F3-1 shows a single migrating spot, indicating lower complexity.

Figure S4. (**A**) Chromatogram of subfraction F3-1 purification using a C18 column, yielding two subfractions: F3-1-1 and F3-1-2. (**B**) Inhibitory activity of F3-1-1 and F3-1-2 against PPL at 100 µg mL^{-1}. Data are mean ± SD (n = 3).

Figure S5. Evaluation of the inhibitory effect of fraction F3-1-1 against PPL using olive oil as substrate (long-chain TGs). The enzyme was pre-incubated with various amounts of F3-1-1 for 30 min at 25 °C in the presence of 4 mM NaTDC and colipase (5-fold molar excess). RA (%) was measured at 37 °C. The α_{I50} value was 52 µg.

Figure S6. UPLC-HRMS/MS characterization of fraction F3-1-1 and annotation of cinncassiol A-19 glucoside. (A) Extracted ion chromatogram of fraction F3-1-1 showing the five major detected compounds in positive ion mode. (B) High-resolution mass spectrum and (C) ESI-CID MS/MS spectrum of compound 3 eluting at RT 5.8 min, annotated as cinncassiol A-19 glucoside. The compound was detected as $[M+H]^+$ at *m/z* 545.25 in positive ionization mode, consistent with the molecular formula $C_{26}H_{40}O_{12}$. Characteristic fragment ions are annotated. Chromatographic separation was performed on a Luna Omega C18 column (100 × 2.1 mm, 1.6 µm) using a water/methanol–acetonitrile gradient containing 0.1% formic acid. MS data were acquired on a Q-TOF mass spectrometer in full-scan and data-dependent MS/MS modes.

Figure S1

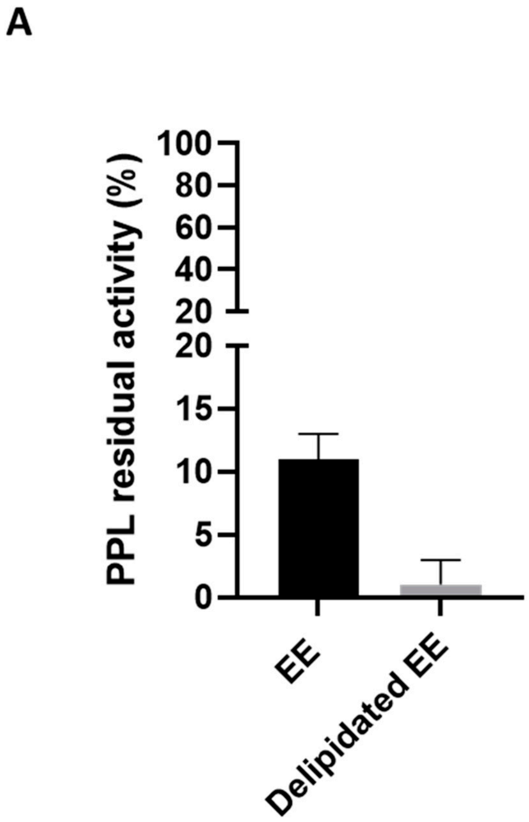
A
PPL residual activity (%)
100
80
60
40
20
20
15
10
5
0
EE
Delipidated EE

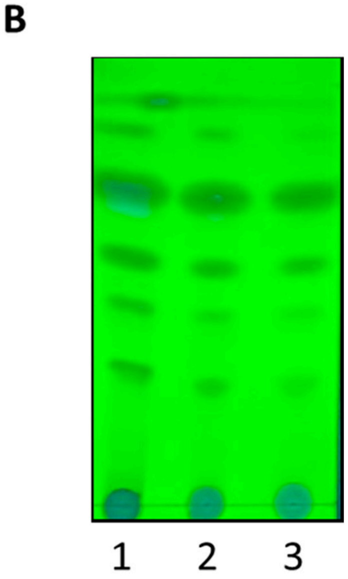
B
1
2
3

Figure S2

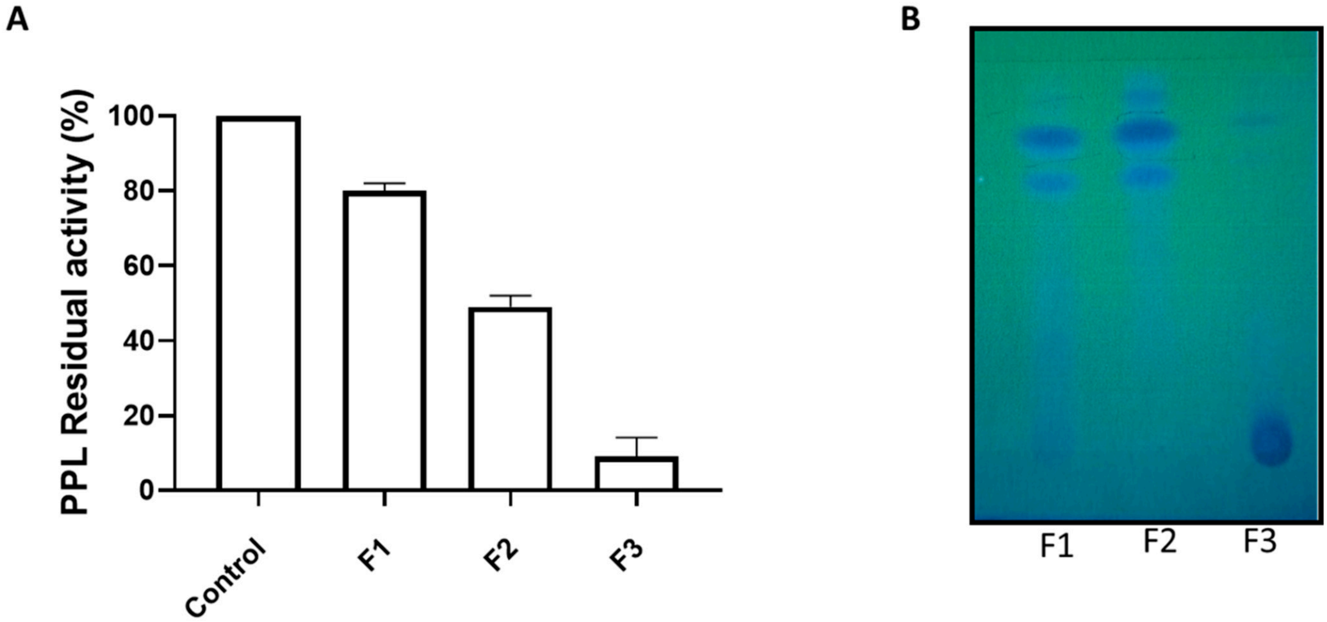
A
PPL Residual activity (%)
100
80
60
40
20
0
Control
F1
F2
F3
B
F1
F2
F3

Figure S3

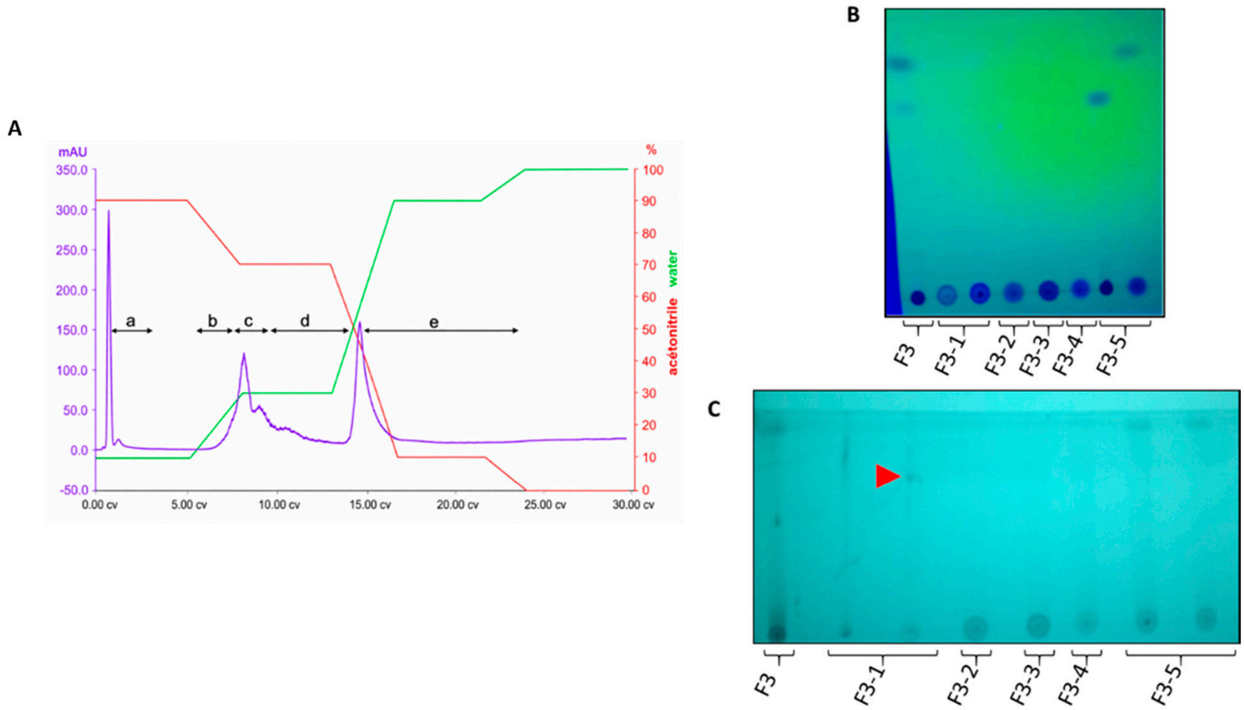
A
mAU
%
350.0
300.0
250.0
200.0
150.0
100.0
50.0
0.0
-50.0
100
90
80
70
60
50
40
30
20
10
0
acétonitrile
water
a
b
c
d
e
0.00 cv
5.00 cv
10.00 cv
15.00 cv
20.00 cv
25.00 cv
30.00 cv
B
F3
F3-1
F3-2
F3-3
F3-4
F3-5
C
F3
F3-1
F3-2
F3-3
F3-4
F3-5

Figure S4

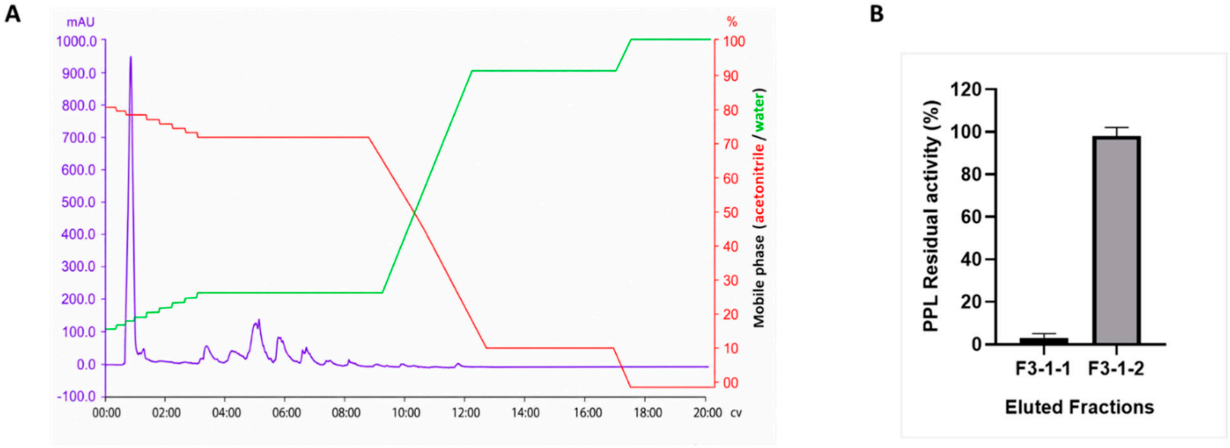
A
mAU
1000.0
900.0
800.0
700.0
600.0
500.0
400.0
300.0
200.0
100.0
0.0
-100.0
00:00
02:00
04:00
06:00
08:00
10:00
12:00
14:00
16:00
18:00
20:00
cv
%
100
90
80
70
60
50
40
30
20
10
00
Mobile phase (acetonitrile / water)
B
PPL Residual activity (%)
120
100
80
60
40
20
0
F3-1-1
F3-1-2
Eluted Fractions

Figure S5

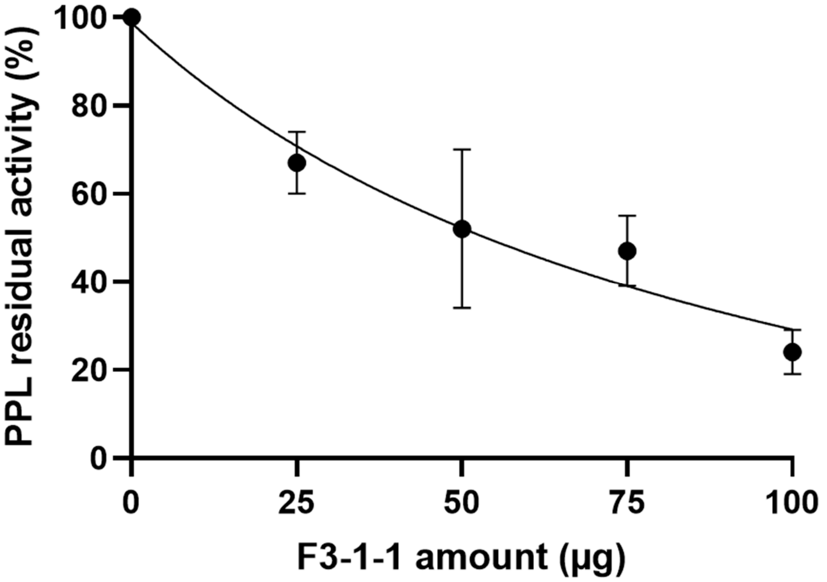

PPL residual activity (%)
100
80
60
40
20
0
0
25
50
75
100
F3-1-1 amount (μg)

Figure S6

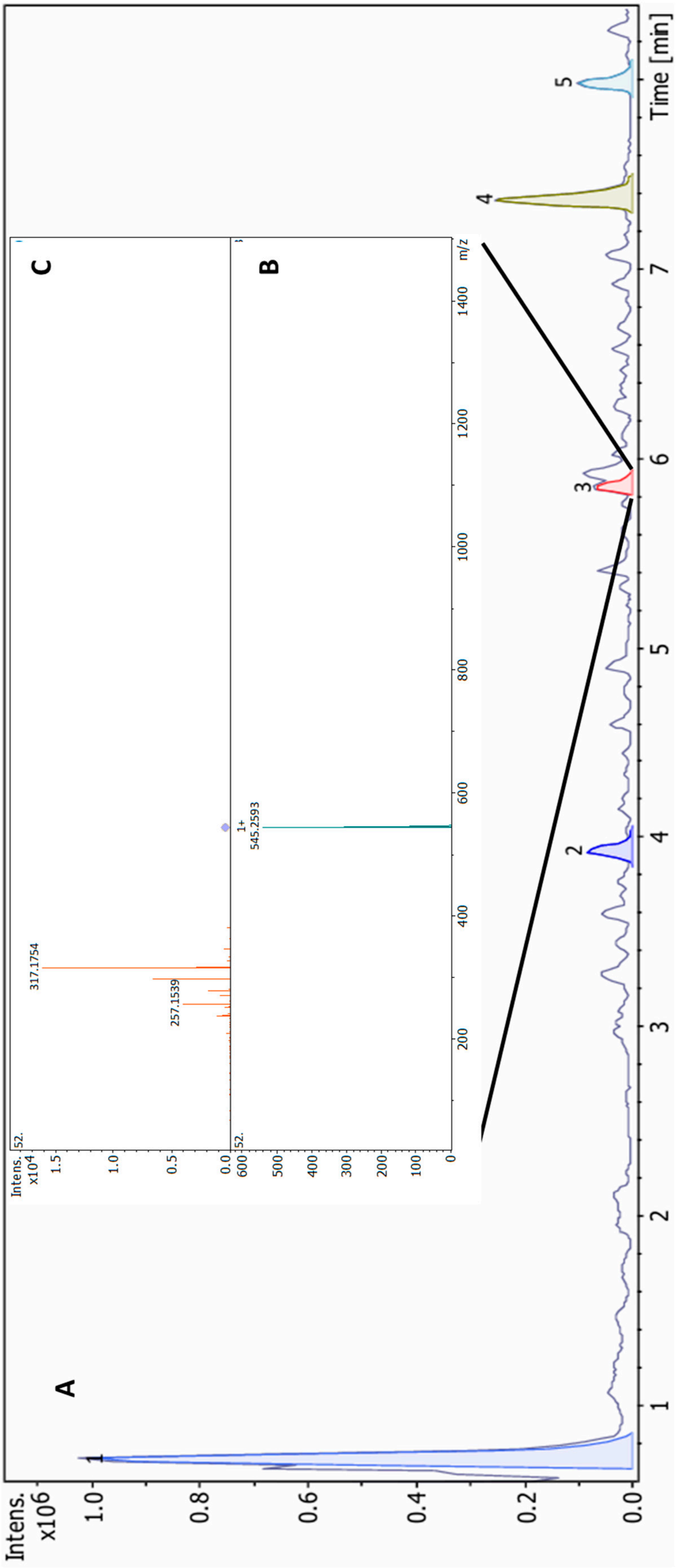

Supplement: Supplementary file 1 [file molecules-31-03737-s001.zip › molecules-4617837-supplementary.pdf]
